# Post-pancreaticoduodenectomy hemorrhage: DSA diagnosis and endovascular treatment

**DOI:** 10.18632/oncotarget.17450

**Published:** 2017-04-27

**Authors:** Tan-Yang Zhou, Jun-Hui Sun, Yue-Lin Zhang, Guan-Hui Zhou, Chun-Hui Nie, Tong-Yin Zhu, Sheng-Qun Chen, Bao-Quan Wang, Wei-Lin Wang, Shu-Sen Zheng

**Affiliations:** ^1^ Hepatobiliary and Pancreatic Interventional Treatment Center, Division of Hepatobiliary and Pancreatic Surgery, Key Laboratory of Combined Multi-Organ Transplantation, Ministry of Public Health, Key Laboratory of Precision Diagnosis and Treatment for Hepatobiliary and Pancreatic Tumor of Zhejiang Province, Zhejiang Province, Hangzhou 310003, China; ^2^ Collaborative Innovation Center for Diagnosis Treatment of Infectious Diseases, Zhejiang University, Hangzhou, Zhejiang, 310003, China

**Keywords:** pancreatoduodenectomy, postoperative hemorrhage, selective angiography, arterial embolism, treatment

## Abstract

**Objective:**

To explore the diagnostic value of digital subtraction angiography (DSA) and the effectiveness of endovascular treatment for a post-pancreaticoduodenectomy hemorrhage (PPH).

**Results:**

During the DSA examination, positive results were found in 29 patients, yielding a positive rate of 69.0%. The manifestations of the DSA examination included contrast medium extravasation, pseudoaneurysm, and artery walls coarse. All 29 patients with positive results underwent endovascular treatment, including transartery embolization (TAE) in 28 patients and covered stents placement in one patient. The technical success and clinical success rates were 100% and 72.4%, respectively. Re-bleeding occurred in 8 of the 29 patients after the first treatment (27.6%). The mortality of PPH was 17.2% (5 of 29). Two of the five PPH patients died following severe infections, and three died from multiple organ failure.

**Materials and Methods:**

A DSA examination was conducted using clinical and imaging data of 42 patients, and endovascular treatment for delayed PPH was retrospectively analyzed.

**Conclusions:**

DSA examination is a minimally invasive and rapid method for the diagnosis of delayed PPH. For patients with positive DSA results, endovascular treatment can be performed rapidly, safely, and effectively. Therefore, the DSA examination and endovascular treatment could be considered a preferred treatment approach for delayed PPH.

## INTRODUCTION

A pancreaticoduodenectomy (PD) is a classic method of operation in the field of abdominal surgery. The perioperative mortality rate for PD has decreased to 5% due to the use of appropriate surgical indications and improved surgical techniques and perioperative management [1]; however, because the method involves multiple organs, the associated procedures are considerably complex, and the incidence of postoperative complications remains at 30%–65% [2]. A post-pancreaticoduodenectomy hemorrhage (PPH) is a less common but potentially fatal complication. Based on the time of occurrence, PPH can be classified as an early hemorrhage, which is frequently a result of technical failure and a delayed hemorrhage that may stem from an ulcer, pseudoaneurysm, eroded vessel, or anastomotic leakage. Endoscopy therapy is used for early upper digestive tract mild hemorrhages; however, angiography interventions or surgical treatments will always be required for delayed hemorrhages. Digital subtraction angiography (DSA) and endovascular treatment are the first choice for the diagnosis and therapy of delayed PPH [4, 5]. For this study, the aim was to evaluate the diagnostic value of DSA as well as the safety, efficacy, and utility of endovascular treatment for delayed PPH.

## RESULTS

The mean duration of PPH was 21 days (range 4–156 d). PPH was clinically suspected in all cases based on acute bleeding into the abdominal drains (*n* = 19), the nasogastric tubes (*n* = 10), or both (*n* = 5). Patients with hematemesis (*n* = 5) and melena (*n* = 3) were also suspected. The angiographic findings of the visceral arteriography are summarized in Table 1.

**Table 1:** Characteristics of DSA examination and endovascular treatment

**Abnormality**	No. (%)
Pseudoaneurysm	1 (3.4%)
Extravasation	22 (75.9%)
Pseudoaneurysm and extravasation	2 (6.9%)
Luminal irregularity	4 (13.8%)
**Origin of bleeding**	
Gastroduodenal artery stump	11 (37.9%)
Superior mesenteric artery	2 (6.9%)
Common/proper hepatic artery	8 (27.6%)
Splenic artery	1 (3.4%)
Right hepatic artery	3 (10.3%)
Left hepatic artery	2 (6.9%)
LGA	2 (6.9%)
Materials and technique	
Coil embolization	15 (51.7%)
Covered stent	1 (3.4%)
Coil embolization and PVA	3 (10.3%)
Coil embolization and gelatin sponge particle	5 (17.2%)
Gelatin sponge particle	5 (17.2%)

**Figure 1:**
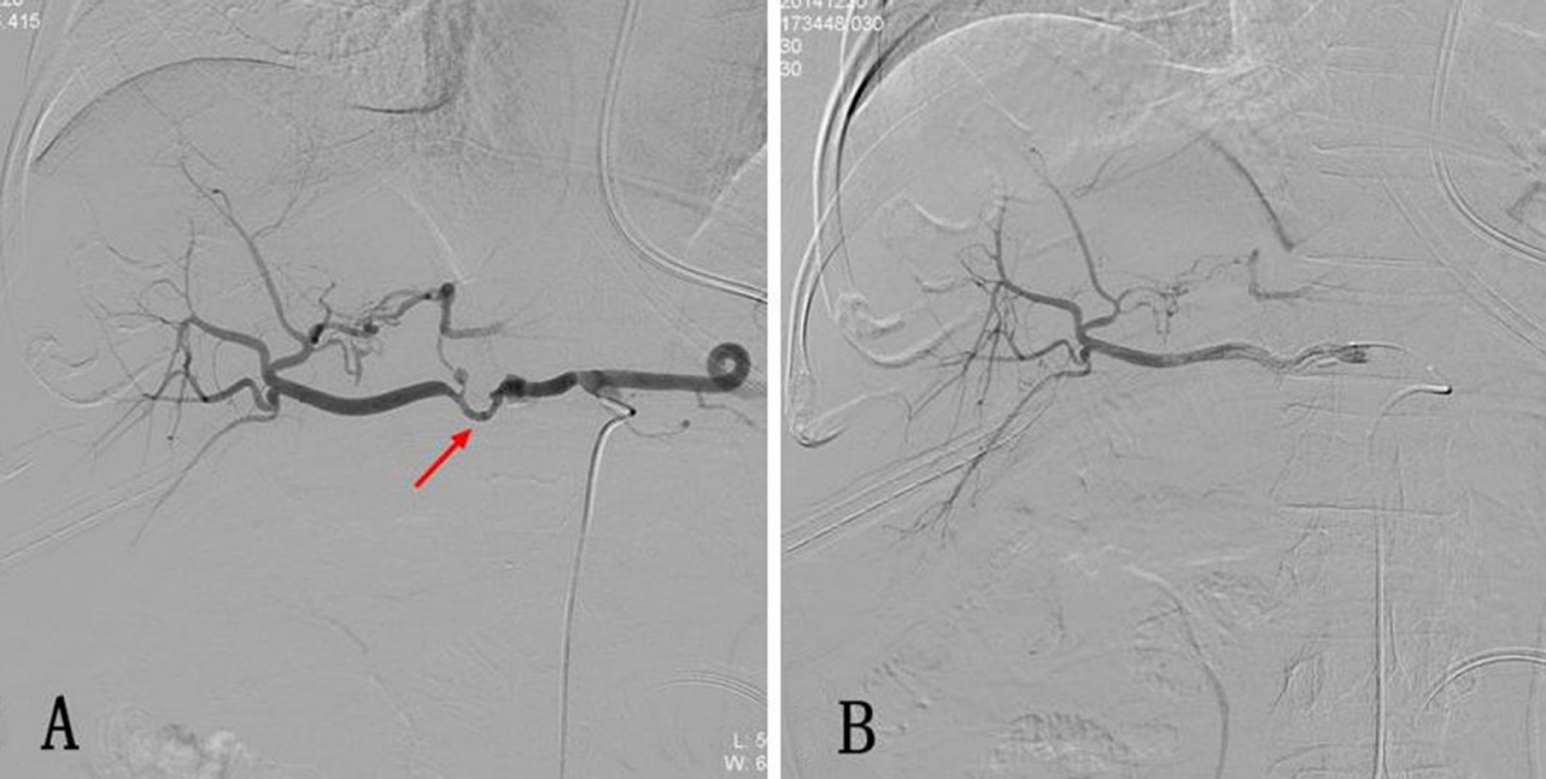
**(A)** Postoperative hemorrhage after Whipple operation. The selective celiac artery arteriography shows the bulging of the proximal end of the common hepatic artery, while the distal end of the artery remains slender. Local overflow of the contrast agent is also displayed (arrow). **(B)** Arteriography was performed again after a 5 mm* 5 cm covered stent was implanted through the vessel lumen, and it shows that the sign of contrast agent overflow disappeared.

**Figure 2:**
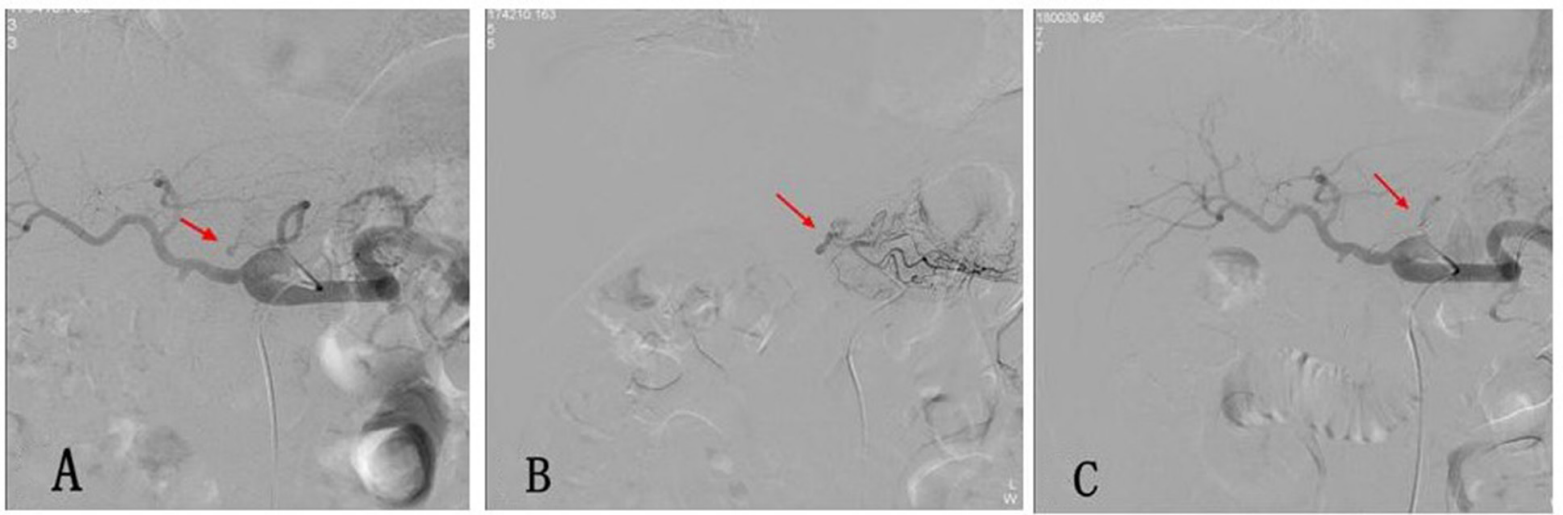
**(A)** Postoperative hemorrhage after Whipple operation. The selective celiac artery arteriography shows the overflow of the contrast agent at the local left gastric artery (arrow). **(B)** The super-selective angiography of the left gastric artery with a 2.7 F micro-catheter shows the local overflow of the contrast agent (arrow). **(C)** A micro-spring steel ring was used for the embolization of the proximal end of the left gastric artery, and a re-examination using an angiography was performed, which revealed signs that the contrast agent overflow disappeared.

All DSA procedures were technically successful, and initial hemostasis was achieved in 29 patients. Of the 29 patients, pseudoaneurysm alone was observed in one patient (3.4%), pseudoaneurysm and active extravasation in two (6.9%), active extravasation in 22 (75.9%), and luminal irregularity in four (13.8%). The hemorrhagic focus in the angiograms was observed in the gastroduodenal artery (GDA) stump in 11 patients, in the hepatic artery in 13, in the superior mesenteric artery in two, in the left gastric artery in two (Figure 2), and in the splenic artery in one. A pushable coil (selective artery embolization) was used as the embolizing agent in 15 patients, and a covered stent was used for one patient (Figure 1). Embolization was established with a pushable coil along with PVA in three cases, a pushable coil along with a gelatin sponge particle in five cases, and a gelatin sponge particle alone in five cases. Recurrent bleeding occurred in eight of the 29 patients (27.6%) after the first endovascular treatment. A repeat procedure was performed in five patients, though the bleeding sites were not discovered, and three patients underwent a laparotomy following endovascular procedures for a washout of a massive hemoperitoneum performed at the discretion of the surgical team (Table 2). Five of the 13 patients with a negative DSA examination changed to symptomatic supportive treatment, and the other eight patients were evaluated using an endoscopy (*n* = 4, 1 with capsule endoscopy) and a laparotomy (*n* = 4) to identify the source of hemorrhage. No procedure-related complication developed in any other case. No rebleeding in a late stage occurred in the other cases. Mortality occurred in five (17.2 %) of the 29 patients: two died from severe infections and three from multiple organ failure.

**Table 2:** Characteristics of patients with recurrent hemorrhages

NO.	Primary bleeding sites and treatment	Recurrence bleeding sites and interval (d)†	treatment
1	PHA(coils)	pancreaticoduodenal anastomosis (3)	emergency exploratory laparotomy and suture hemostasis
2	GDA(coils)	NA (1)	medicine treatment
3	CHA(coils)	gastrojejunal anastomosis (4)	emergency exploratory laparotomy and suture hemostasis
4	GDA(coils)	NA (0.5)	medicine treatment
5	CHA(coils)	NA (1)	medicine treatment
6	GDA (coils& PVA)	NA (2)	medicine treatment
7	GDA(coils)	gastrojejunal anastomosis (10)	emergency exploratory laparotomy and suture hemostasis
8	PHA(coils)	NA (2)	medicine treatment

## DISCUSSION

A postoperative hemorrhage is a uncommon complication after PD but is one of the major causes of peri-operative mortality. The incidence of postoperative hemorrhage is about 4%–16%, and the mortality is as high as 11%–54% [5]. Based on the time frame in which a hemorrhage occurs after PD, hemorrhages can be classified as early (within 24 h after PD) or delayed hemorrhages (after 24 h after PD), while based on the site of a hemorrhage, it can be classified as gastrointestinal or intra-abdominal [6]. In this study, all 42 patients suffered from delayed hemorrhages. Early hemorrhages after PD are generally directly associated with the surgical procedure, such as an incomplete hemostasis during an operation and loose anastomosis, while delayed hemorrhages are generally caused by abdominal infections due to anastomotic fistula, tissue necrosis and sloughing, and erosion and bleeding of the wound surface or vessel [7], such as pseudoaneurysm and stomal ulceration. In this study, pancreatic fistula was confirmed in four patients and pseudoaneurysm in three. In addition, ulcer gastrojejunal anastomosis was confirmed by endoscopy in two patients with a negative DSA examination.

In clinical practice, the most commonly used diagnostic methods for PPH include an abdominal CT, gastrointestinal endoscopy, and DSA. DSA is currently recommended as the first line of care in patients with PPH because it can directly diagnose a hemorrhage [8], clearly identify its site, and assist with endovascular treatment. If endovascular treatment fails, the results of the DSA still provide important values for consequent surgical treatment [9]. Therefore, the DSA has been widely favored in the clinical diagnosis and treatment of various postoperative hemorrhages [10, 11]. Previous studies have shown that the DSA can correctly position 70–90% of arterial hemorrhages, while the intermittence of hemorrhages is a possible cause of a false negativity of a DSA [12] (Table 3). Some researchers have therefore suggested that if a DSA examination fails to clarify the hemorrhage site, a repeated DSA can be performed 6–24 h later in patients with stable hemodynamics [13].

**Table 3:** Published series of endovascular treatment for PPH that included > 10 patients during a 6-year period

References	Published Year	Study Design	Patients (Male/Female)	Mean Age (Y)	Technical Success	Clinical Success	Recurrent Bleeding	Mortality Rate	Major Complications	Embolization Technique
Hasegawa T, [19]	2017	retrospective study	28 (25/3)	64.7 (range 40–82)	100%	75% (21/28)	25% (7/28)	28.6%(8/28)	9 (32.1%) Hepatic failure (7) Hepatic abscess (4) Both (2)	microcoils (*n =* 17) covered stents (*n =* 1) bare stent-assisted coil embolization (*n =* 5) catheter grafts with coil embolization (*n =* 5)
Hassold N, [20]	2016	retrospective study	27 (23/4)	60.8 (range 39–85)	stent graft group 88% (14/16) embolization group 91% (10/11)	stent graft group 81%(13/16) embolization group 100%(11/11);	stent graft group 12.5 (2/16) embolization group 0 % (0/11)	stent graft group 19% embolization group 30 %	stent graft group 56.3%(9/16) Intestinal ischemia (1) Multiorgan failure (4) Rebleeding (2) Rupture of pseudoaneurysm (1) Hepatic abscess (1) embolization group 54.5%(6/11) Hepatic failure (3) Spleen infarction (1) Hepatic abscess (2)	covered stents (*n =* 12) coils embolization (*n =* 9) covered stent-assisted coil embolization (*n =* 1) covered stent and Amplatzer plug (*n =* 1) Amplatzer plug (*n =* 1)
Gaudon C, [8]	2016	retrospective study	42 (27/15)	61.8 (range 19–81)	subgroup of anatomical variation:37.5% subgroup of modal anatomy:82.8%	85% (29/34)	NA	subgroup of anatomical variation:36.4% subgroup of modal anatomy:6.5%	12% liver abscess or liverischemia. (*n =* 5)	Covered stent (*n =* 16) Selective coil embolization of gastroduodenal artery (*n =* 4) Common hepatic artery embolization (*n =* 5)
Ching, KC, [21]	2016	retrospective study	28 (23/5)	65.1 (range,40–86).	97.4% (37/38)	73.7% (28/38)	26.3% (10/38)	7.1% (2/28)	NA	covered stents (*n =* 25) coils Embolization (*n =* 9) covered stent-assisted coil embolization (*n =* 2) balloon angioplasty(*n =* 1).
Huo Y, [3]	2015	retrospective study	21 (17/4)	63.5 (range 36–80)	100%(18/18)	77.8% (14/18)	22.2% (4/18)	22.2% (4/18)	50% (9/18) re-bleeding (*n =* 4) liver damage (*n =* 3) liver abscess (*n =* 2)	transcatheter arterial embolization (*n =* 10) covered stent placement (n = 8) laparotomy (*n =* 3)
Adam G, [22]	2014	retrospective study	16 (12/4)	58.0 (range 39–79)	100%	100%	6.3% (1/16)	18.7% (3/16)	NA	pushable coil (*n =* 13) pushable coil +NBCA (*n =* 2) covered stent (*n =* 1)
Stampfl U, [23]	2012	retrospective study	25 (19/6)	59 (range 29–73)	83% (19/23)	78.3% (18/23)	16% (3/19)	20%	NA	Coil embolization alone (*n =* 7) liquid embolization alone (*n =* 2) combination of coil and liquid embolization (*n =* 11) stent and coils (*n =* 1)
Hur S, [24]	2011	retrospective study	16 (13/3)	73 (range, 64–88)	100%	NA	18.8% (3/16)		31.3% (5/16) Multiple organ failure (*n =* 2) subsegmental liver infarction (*n =* 1) multisegmental liver infarction (*n =* 2,)	Trapping HA (n = 13) Embolization of GDA (*n* = 3)
Zhang J, [25]	2011	retrospective study	14 (10/4)	58.9 (range, 25–73)	100%	85.70%	NA	28.6% (4/14)	NA	coils Embolization (*n =* 14)

The number of patients per study, mean patient age, number of patients who underwent endoscopy before embolization, proportion of the patients who were found to have active extravasation on initial angiography, and technical success rate of embolization are presented.

NA not available.

The overflow of a contrast agent is a direct sign of hemorrhage in a DSA examination; however, a DSA examination can detect signs of a contrast agent overflow when the velocity of the hemorrhage is 0.5–1.0 ml/min. Indirect signs of a post-PD hemorrhage during a DSA examination mainly include the formation of pseudoaneurysm, vasospasm, and unsmooth blood vessel walls. Among these signs, bleeding from the rupture of a pseudoaneurysm is the major cause of delayed hemorrhages [14]. The formation of a pseudoaneurysm is closely associated with injury and thinning of the arterial wall, pancreatic fistula, biliary fistula, and abdominal infection induced by lymph node dissection and separation of a tumor from the vascular wall during an operation. When a patient experiences an elevation in blood pressure or is directly impacted by exogenous forces, the pseudoaneurysm may rupture easily, thus inducing a gastrointestinal or intra-abdominal hemorrhage. In the 42 patients with hemorrhages after PD, 29 had positive DSA results, with a total positive rate of 69%, while 13 patients were negative, which could be associated with a low hemorrhage velocity without signs of a contrast agent overflow or a DSA that was performed at the intermittence of a hemorrhage. Furthermore, the volume of blood loss was relatively high, and blood oxygen was relatively low in these patients, so they could not cooperate well with the examination due to tachypnea, and the DSA images were relatively unclear. These factors could have contributed to a false negative DSA result.

Endovascular treatment has several advantages for PPH, such as being minimally invasive and safe and having a definitive treatment effectiveness. Thus, it has been considered the preferred method for the treatment of post-PD hemorrhages instead of surgical treatment [15]. The embolization materials required for endovascular treatment in clinical practices mainly include a micro-spring steel ring, a PVA particle, and a gelatin sponge (Table 3). A micro-spring steel ring is a permanent embolization material mainly used for the embolization of a pseudoaneurysm and broken end(s) of relatively large arteries. A PVA particle is also a permanent embolization material mainly used for the embolization of hemorrhages of relatively small arteries. While a gelatin sponge is an embolization material with moderate effectiveness that is inexpensive and easily obtained, it is mainly used in combination with other permanent embolization materials to assist in the embolization of various hemorrhages. In addition, a gelatin sponge can also be formed into different shapes according to various surgical requirements. The clinical application of covered stents also provides a new approach for the endovascular treatment of hemorrhages. For the 29 patients with positive results in this study, hemostasis was successful in 21 of the patients, and the success rate of hemostasis was 72.4%. For patients with negative DSA results, transcatheter arterial infusion vasopressin has been suggested because it may sufficiently reduce blood flow to a site of mesenteric bleeding to allow for clot formation; however, it may be ineffective in cases of hemorrhages that originate from a large caliber vessel or in lesions that have a dual blood supply. Moreover, vasopressin should not be used to treat patients with coronary artery disease, severe hypertension, cardiac arrhythmias, or limb ischemia. Vasopressin therapy for a gastrointestinal hemorrhage has been replaced almost completely by superselective transcatheter embolotherapy [16]. Thus, the 13 patients with negative results did not adopt infusion vasopressin to hemostasis and underwent other treatments.

In eight patients in this study, the DSA examination showed that the site of hemorrhage was at the initiation of the hepatic artery in five patients and at the proper hepatic artery in three. One patient received covered stent implantation treatment, and the other seven patients underwent embolization of the common or proper hepatic arteries via micro-spring steel rings and gelatin sponges after consultations between the clinicians and patients. No liver failure or evident liver necrosis was found in the seven patients after the operations. Mine T et al. [17]. speculated that the embolization of the proper hepatic artery for hemostasis is safe and effective, especially for patients in whom preoperative liver function is normal and without portal vein stenosis, as embolizing the proper hepatic artery for two weeks could induce blood supply to the liver from extrahepatic collateral branches; however, other researchers also suggested that in patients with hemorrhages after liver, gallbladder, or pancreatic operations, the embolization of the proper hepatic or common hepatic artery could induce ischemic injuries of the liver, among which hepatic infarction was the most common condition [18]. Therefore, further clinical studies are needed to investigate whether the proper hepatic or common hepatic artery should be embolized for the treatment of post-PD hemorrhages with endovascular treatment; however, in recent years, the application of covered stents has also provided a new management approach for the treatment of hemorrhages from the proper hepatic or common hepatic arteries, which could ensure arterial perfusion and help restore liver functions in addition to establishing hemostasis.

There are few complications involved in treating post-PD hemorrhages via endovascular modalities. Potential complications mainly include intestinal ischemia and necrosis, liver damage, hepatic infarction, infection, and ectopic embolism. Generally, researchers have suggested that super-selectively catheterizing the hemorrhagic site and then embolizing the site during operation could effectively prevent or reduce the incidence of complications. In addition, active postoperative symptomatic treatment could also help prevent and reduce the incidence of complications. In this study, transient liver dysfunction was found in 19 patients who underwent endovascular treatment, with the primary manifestation of elevated levels of transaminase. Sixteen patients suffered from fever, which was alleviated after antibiotic treatment and physical cooling was initiated, and five patients suffered from abdominal pain and melena, which disappeared after symptomatic treatment.

There are several limitations to this study. First, the sample size was relatively small. Second, this study was retrospective and lacked randomization. Although prospective randomization would have been helpful to assess the outcome of endovascular treatment in comparison with an endoscopy or laparotomy, it is difficult from an ethical point of view to perform a randomized controlled study in life-threatening situations. Third, follow-up periods differ significantly in the study population. A future prospective study with a standardized treatment and follow-up algorithm would be required to determine the benefits of one treatment technique versus another.

In summary, a postoperative hemorrhage post-PD is one of the most serious complications in clinical practice. Conducting a DSA examination in a timely manner when conservative treatment fails could help clarify the causes and sites of hemorrhages, of which the common positive signs mainly include an overflow of the contrast agent and pseudoaneurysm formation. Choosing appropriate embolization materials or using covered stents for endovascular treatment of the hemorrhagic artery could result in ideal hemostatic effectiveness. In addition, these treatment modalities are safe and have a low risk of complications. Therefore, these findings suggest that DSA examinations and endovascular treatment could be the preferred diagnostic and treatment methods for postoperative hemorrhages after PD.

## MATERIALS AND METHODS

### Patients

The data were obtained from a retrospective clinical database and reviewed for patients undergoing angiography for suspected late PPH between March 2013 and June 2016. Institutional Review Board approval was waived for this retrospective study. Due to the retrospective nature of this study, informed consent from patients was not required. There were 42 patients (31 male, 11 female; mean age 61.6 ± 8.6 years; range 42–81 years) identified who had undergone diagnostic angiography and endovascular treatment including covered stent implantation and embolization due to delayed PPH. The different characteristics of the population were recorded prospectively in the database and are summarized in Table 4.

**Table 4:** Patient characteristics of PPH

**Age(year)**	No.(%)	**Diagnosis**	No. (%)
Mean	61.6 ± 8.6	Hilar cholangiocarcinoma	3 (7.1%)
Range	[42–81]	Pancreatolithiasis	1 (2.4%)
**Gender**		Pancreatic metastases form colon cancer	1 (2.4%)
Male	31 (73.8%)	**Postoperative Interval (d)**	
Female	11 (26.2%)	Mean	21.1 ± 24.8
**Diagnosis**		Range	[3–156]
Pancreatic cancer	15 (35.7%)	**Presentation of SB**	
Ampullary cancer	4 (9.5%)	Abdominal drains	19 (45.2%)
Distal common bile duct carcinoma	6 (14.3%)	Nasogastric tubes	10 (23.8%)
Duodenal papillary carcinoma	7 (16.7%)	Abdominal drains and Nasogastric tube	5 (11.9%)
Gallbladder carcinoma hilar metastasis	4 (9.5%)	Haematemesis	5 (11.9%)
Hepatolithiasis	1 (2.4%)	Melena	3 (7.1%)

### Methods

#### Diagnostic angiography

The angiography was performed using the Allura Xper FD20 system (Philips, Amsterdam, the Netherlands). For vascular access, a 5-F sheath was introduced in the right femoral artery. In all patients, selective and superselective angiograms were obtained, including the late (portal venous) phase. A selective angiography was performed using a 5-F RH catheter (Terumo, Tokyo, Japan) positioned in the celiac trunk and in the superior mesenteric artery with an automated injection of 35 mL of iodinated contrast material at a rate of 5–6 mL/s with 300 psi of pressure. A superselective angiography was performed with an automated injection of 2.5 mL of iodinated contrast material via a 2.8-F microcatheter system (Renegade Hi-Flo Fathom^™^; Boston Scientific, Natick, USA) with 500 psi of pressure. The definition and evaluation of the arterial hemorrhage for an angiography was identical to a multidetector CT and was separately analyzed using selective and superselective angiography. Similar to multidetector CT imaging, the angiography was reviewed by two experienced radiologists (SUN, ZHOU.), and decisions were made based on consensus.

### Endovascular treatment

Endovascular treatment wascarried out for patients with angiographically proven arterial hemorrhage. Embolization was performed with a superselective catheter position by coil embolization (fibered platinum coils and Interlocking Detachable Coils; Boston Scientific, Cork, Ireland), polyvinyl alcohol (PVA) particles (diameter of 710–1000 μm; Ailikang, Hangzhou, China), or gelatin sponge (Jinling Pharmaceutical Co. Ltd., Nanjing, China). The techniques used for coil embolization included distal and proximal embolization to exclude the abnormality, embolization of a vascular stump created during surgery, embolization of distal right or left hepatic arteries, or direct coil embolization of pseudoaneurysms if no other technique was suitable. The coils were placed until complete exclusion of the bleeding site was obtained, which was defined by the absence of the vascular abnormality on the repeat angiography. PVA or gelatin sponge embolization was most often performed for lower gastrointestinal (GI) hemorrhages that originated from the branches of superior mesenteric artery(SMA) or for coil-assisted embolization. Covered stents were placed for vascular injuries of the common or proper hepatic artery, SMA, and the splenic artery to maintain distal organ perfusion. A 45-cm, a 6–9- F curved reinforced sheath (Flexor Ansel; Cook) was placed at the celiac or SMA origin for the delivery of stent grafts. Self-expanding nitinol/expanded polytetrafluoroethylene (VIABAHN; W.L. Gore & Associates, Flagstaff, Arizona) stent grafts of 5–10 mm in diameter were used. Antiplatelet agents were not administered after stent graft placement. Technical success was defined as a complete occlusion of the bleeding site or pseudoaneurysm with the absence of contrast material extravasation shown by selective and superselective control angiographies 15 minutes after embolization.

### Effectiveness evaluation criteria

The effectiveness, technical success, and clinical success of endovascular treatment for a hemorrhage after PD were defined and evaluated according to the Society of Interventional Radiology (SIR) guidelines. The detailed definitions are as follows:

Technical success: re-examination of angiography immediately after endovascular treatment shows the hemorrhagic artery was blocked with no direct or indirect signs of hemorrhage;

Clinical success: hemodynamics remain stable within 30 days after endovascular treatment, the level of hemoglobin increases, and no re-occurrence of hemorrhage is noted. Clinical success can be further divided into complete success and partial success. Complete success is defined as syndromes or symptoms of hemorrhage that completely disappear, while partial success is defined as syndromes or symptoms of hemorrhage that improve,disease conditions are temporarily stabilized, which allows for further clinical treatments.

### Follow-up

Patients were examined for clinical symptoms of ongoing or recurrent hemorrhages. The patients’ clinical conditions and laboratory test results (hemoglobin concentration, lactate level, liver enzymes) were documented until discharge or death as well as during the appointments at the outpatient clinic up to the recorded period. Complications and mortality that occurred during the course of the study were documented. Recurrent bleeding and ischemic effects were assessed by follow-up CT scans, or in the case of clinical symptoms of acute rebleeding, by angiography. The mean imaging follow-up time was 23 days (range, 6–81 days). The mean clinical follow-up time was 107 days (range, 35–225 days).
