# Correction: Post-pancreaticoduodenectomy hemorrhage: DSA diagnosis and endovascular treatment

**DOI:** 10.18632/oncotarget.24760

**Published:** 2018-03-16

**Authors:** Tan-Yang Zhou, Jun-Hui Sun, Yue-Lin Zhang, Guan-Hui Zhou, Chun-Hui Nie, Tong-Yin Zhu, Sheng-Qun Chen, Bao-Quan Wang, Wei-Lin Wang, Shu-Sen Zheng

**Affiliations:** ^1^ Hepatobiliary and Pancreatic Interventional Treatment Center, Division of Hepatobiliary and Pancreatic Surgery, Key Laboratory of Combined Multi-Organ Transplantation, Ministry of Public Health, Key Laboratory of Precision Diagnosis and Treatment for Hepatobiliary and Pancreatic Tumor of Zhejiang Province, First Affiliated Hospital, College of Medicine, Zhejiang University, Zhejiang Province, Hangzhou 310003, China; ^2^ Collaborative Innovation Center for Diagnosis Treatment of Infectious Diseases, Zhejiang University, Hangzhou, Zhejiang, 310003, China

**This article has been corrected:** The proper name of the institution is as follows:

^1^ Hepatobiliary and Pancreatic Interventional Treatment Center, Division of Hepatobiliary and Pancreatic Surgery, Key Laboratory of Combined Multi-Organ Transplantation, Ministry of Public Health, Key Laboratory of Precision Diagnosis and Treatment for Hepatobiliary and Pancreatic Tumor of Zhejiang Province, First Affiliated Hospital, College of Medicine, Zhejiang University, Zhejiang Province, Hangzhou 310003, China

Original article: Oncotarget. 2017; 8:73684-73692. https://doi.org/10.18632/oncotarget.17450

